# Smoking status and clinical outcome in idiopathic pulmonary fibrosis: a nationwide study

**DOI:** 10.1186/s12931-024-02819-w

**Published:** 2024-04-29

**Authors:** Hee-Young Yoon, Hoseob Kim, Yoonjong Bae, Jin Woo Song

**Affiliations:** 1 Division of Allergy and Respiratory Diseases, Soonchunhyang University Seoul Hospital, Seoul, Republic of Korea; 2 Department of Data Science, Hanmi Pharm. Co., Ltd, Seoul, Republic of Korea; 3 Department of Pulmonary and Critical Care Medicine, Asan Medical Center, University of Ulsan College of Medicine, 88, Olympic-Ro 43-Gil, Songpa-Gu, Seoul, 05505 Republic of Korea

**Keywords:** Idiopathic pulmonary fibrosis, Smoking, Mortality, Hospitalization, Prognosis

## Abstract

**Background:**

Smoking status has been linked to the development of idiopathic pulmonary fibrosis (IPF). However, the effect of smoking on the prognosis of patients with IPF is unclear. We aimed to investigate the association between smoking status and all-cause mortality or hospitalisation by using national health claims data.

**Methods:**

IPF cases were defined as people who visited medical institutions between January 2002 and December 2018 with IPF and rare incurable disease exempted calculation codes from the National Health Insurance Database. Total 10,182 patients with available data on smoking status were included in this study. Ever-smoking status was assigned to individuals with a history of smoking ≥ 6 pack-years. The multivariable Cox proportional hazard model was used to evaluate the association between smoking status and prognosis.

**Results:**

In the entire cohort, the mean age was 69.4 years, 73.9% were males, and 45.2% were ever smokers (current smokers: 14.2%; former smokers: 31.0%). Current smokers (hazard ratio [HR]: 0.709; 95% confidence interval [CI]: 0.643–0.782) and former smokers (HR: 0.926; 95% CI: 0.862–0.996) were independently associated with all-cause mortality compared with non-smokers. Current smokers (HR: 0.884; 95% CI: 0.827–0.945) and former smokers (HR: 0.909; 95% CI: 0.862–0.959) were also associated with a reduced risk of all-cause hospitalisation compared with non-smokers. A non-linear association between smoking amount and prognosis was found in a spline HR curve and showed increasing risk below 6 pack-years.

**Conclusion:**

Ever-smoking status may be associated with favourable clinical outcomes in patients with IPF.

**Supplementary Information:**

The online version contains supplementary material available at 10.1186/s12931-024-02819-w.

## Background

Idiopathic pulmonary fibrosis (IPF) is a chronic progressive fibrosing interstitial pneumonia of unknown aetiology predominantly affecting older male smokers [1]. Recent studies suggest that recurrent injuries to the alveolar epithelium stimulate the fibrogenic pathway, thus leading to the activation of fibroblasts and the production of excessive extracellular matrix in genetically susceptible individuals [2]. Risk factors for the IPF include older age, male sex, genetic mutation, environmental and occupational exposures [3–6], and cigarette smoking [6–11].

Previous studies have highlighted the association between cigarette smoking and IPF development [7, 10, 12–14], with 60–80% of IPF having a history of smoking [12–14]. Recent studies showed a dose-dependent relationship between smoking amount and IPF incidence [7, 10]. However, the effect of smoking on IPF prognosis is controversial [15–20]. Some research indicates smokers with IPF have better outcomes than non-smokers [15, 16, 20], whereas others find no difference when considering disease severity [17–19]. A recent study on patients with interstitial lung disease (ILD) (*n* = 377, IPF = 59) showed that heavy smokers (≥ 20 pack-years) had worse survival than never or mild smokers (0.1–19.9 pack-years) [21]. Smoking may also affect poor survival because it increases the risk of lung cancer [22, 23]. However, many of these findings come from single-center studies with limited number of patients (*n* = 98–461) [15–20]. Thus, we aimed to investigate the association between smoking status and prognosis in a large number of patients with IPF by using a nationwide claims database.

## Methods

### Data sources

Data were obtained from the National Health Insurance Sharing Service (NHISS) database, which includes all claims data, such as qualification, insurance premiums, registration status for rare and incurable diseases, clinic visits, and treatment status, of Korean citizens. All South Korean residents aged ≥ 20 years are provided with a biennial health check-up, including smoking status [24], and this information is also stored in the NHISS database. Survival data were obtained from the Korean Statistical Information Service. The Institutional Review Board of Asan Medical Center approved this study (no. S2021-1136-0011) and did not require informed consent due to its retrospective nature and the use of de-identified data.

### Study population

IPF cases were identified using both the IPF diagnostic code of the Korean Standard Classification of Disease (KCD) (7^th^ edition), a modified version of the International Classification of Disease and Related Health Problems (10^th^ revision), and a rare intractable diseases (RID) program code. To be eligible for the RID program, patients must meet the National Health Insurance (NHI) criteria, which require the (1) exclusion of other conditions that could cause ILD, (2) presence of a usual interstitial pneumonia pattern on chest computed tomography (CT) or on surgical lung biopsy along with corresponding chest CT findings. Owing to the strictness of the final registration process reviewed by the NHI, the RID code has been used for identification of other rare diseases in previous studies [25–27].

We screened 22,301 patients who visited secondary and tertiary medical institutions with both IPF (J84.1) and RID registration (V236) diagnostic codes and underwent chest CT within 3 months from the index date (the first date of identification of J84.1 and V236 codes) (Additional file 1: Fig. S1). From these, we excluded those diagnosed in 2018 due to insufficient follow-up (*n* = 2,407), those under 50 years (*n* = 767) considering the lower possibility of IPF diagnosis, and those without a recorded smoking status as they didn’t undergo health check-ups (*n* = 8,948). A total of 10,182 patients were included in this study.

### Definition

The participants were classified into never, former, and current smokers on the basis of their smoking status. Ever smokers (current and former smokers) were defined as individuals with ≥ 6 pack-years of smoking by using spline hazard ratio (HR) curve analysis. Former smokers were defined as individuals who smoked at least 6 pack-years in their lifetime but had quit smoking at survey time [28]. The follow-up periods were calculated from the index date to the occurrence of the events or censoring (December 2018). The primary outcome was the occurrence of all-cause death or the first hospitalisation for all-cause or respiratory cause. Respiratory hospitalisations were identified using codes for diseases of the respiratory system (KCD J00-J99). Comorbidities were identified when patients had ≥ two visits to medical institutions with the same comorbidity codes within 1 year from the index date. Medication history included the use of antifibrotics (pirfenidone) or corticosteroids (oral or injectable form) for ≥ 1 month. The analysis also used socioeconomic variables as covariates, including insurance types (NHI vs. medical aid), household income (high vs. low [defined as the lowest 30% of NHI premium]), and residence type (urban vs. rural areas).

### Statistical analysis

All variables were presented as mean ± standard deviation or numbers (percentage). Differences between groups were assessed using paired t-tests or chi-square tests. Kaplan–Meier survival curve analysis and a log-rank test evaluated survival differences among groups. Cox proportional hazards analysis was performed to identify the risk factors for mortality or hospitalisation. A multivariable analysis was adjusted for preselected covariates, including clinical (age, sex, diagnosis year, Charlson comorbidity index [CCI], prescribed medication, and home oxygen use) and socioeconomic (type of insurance, income, region) covariates. To evaluate the association between smoking amount and prognosis, smoking amount was examined as a continuous variable or by using quartiles: Q1 (1–17 pack-years), Q2 (18–29 pack-years), Q3 (30–39 pack-years), and Q4 (40–200 pack-years). We performed subgroup analyses based on sex (male vs. female) and age (< 65 years vs. ≥ 65 years). We also performed analyses using three different approaches: stratification by quartiles of year of diagnosis (Q1: 2009–2010, Q2: 2011–2012, Q3: 2013–2014, Q4: 2015–2017), antifibrotics availability (before vs. after October 2015), and treatment status (none, antifibrotics only, steroids only, antifibrotics and steroids). A cubic spline HR curve analysis was used to identify the non-linear dose-dependent relationships after adjusting for clinical and socioeconomic covariates. The adjusted HR used 6 pack-years (the lowest HR value observed in the spline curve analysis) as a reference. Analyses were conducted using SAS version 9.4 (SAS Institute, Cary, NC, USA), and a two-tailed *p*-value of < 0.05 was considered statistically significant.

## Results

### Baseline characteristics and outcomes

Among the patients (*n* = 10,182), the mean age was 69.4 years, 73.9% were male, and 45.3% were ever smokers (current: 14.2%; former: 31.0%) (Table 1). The mean smoking amount was 10.6 ± 12.8 pack years, and Fig. S2 in Additional file 1 shows participant distribution of smoking amounts. The most common comorbidity was dyslipidaemia (69.6%), followed by hypertension (59.4%).

**Table 1:** Comparison of baseline characteristics of patients with IPF according to smoking status

	Total	Never	Ever smokers		
			Total	Former	Current
Number of patients	10,182	5,574	4,608	3,159	1,449
Age	69.4 ± 8.1	70.4 ± 8.3*^†‡^	68.4 ± 7.8	69 ± 7.7^§^	66.7 ± 7.5
Male	7,528 (73.9)	3,011 (54.0)*^†‡^	4,517 (98.0)	3,129 (99.1)^§^	1,388 (95.8)
Body mass index, kg/m^2^	^‡^24.0 ± 3.0	23.9 ± 3.1^†‡^	24.0 ± 3.0	24.2 ± 2.9^§^	23.7 ± 3.0
Smoking amount, pack years	10.6 ± 12.8	0.0 ± 0.0*^†^	23.5 ± 7.8	23.3 ± 7.9	23.8 ± 7.8
Low household income	1,528 (15.0)	835 (15.0)^‡^	693 (15.0)	451 (14.3)^§^	242 (16.7)
Medical aid	119 (1.2)	56 (1.0)^‡^	63 (1.4)	28 (0.9)^§^	35 (2.4)
Comorbidity					
Lung cancer	1,134 (11.1)	551 (9.9)*^†‡^	583 (12.7)	383 (12.1)^§^	200 (13.8)
Diabetes mellitus	5,285 (51.9)	2,865 (51.4)^†^	2,420 (52.5)	1,702 (53.9)^§^	718 (49.6)
Dyslipidaemia	7,084 (69.6)	3,931 (70.5)*^‡^	3,153 (68.4)	2,209 (69.9)^§^	944 (65.1)
Hypertension	6,047 (59.4)	3,346 (60.0)	2,701 (58.6)	1,906 (60.3)^§^	795 (54.9)
Ischaemic heart disease	3,174 (31.2)	1,730 (31.0)	1,444 (31.3)	1,031 (32.6)^§^	413 (28.5)
Arrhythmias	1,198 (11.8)	650 (11.7)	548 (11.9)	409 (12.9)^§^	139 (9.6)
Infection	1,849 (18.2)	1,076 (19.3)*^†‡^	773 (16.8)	556 (17.6)^§^	217 (15.0)
Tuberculosis	1,743 (17.1)	1,020 (18.3)*^†‡^	723 (15.7)	520 (16.5)^§^	203 (14.0)
NTM^§^	94 (0.9)	60 (1.1)^‡^	34 (0.7)	27 (0.9)	7 (0.5)
Fungal infection	80 (0.8)	42 (0.8)	38 (0.8)	31 (1.0)^§^	7 (0.5)
Invasive pulmonary aspergillosis	98 (1.0)	51 (0.9)	47 (1.0)	36 (1.1)	11 (0.8)
COPD	1,241 (12.2)	590 (10.6)*^†‡^	651 (14.1)	457 (14.5)^§^	194 (13.4)
Renal failure	810 (8.0)	441 (7.9)	369 (8.0)	273 (8.6)^§^	96 (6.6)
CCI	1.3 ± 0.9	1.3 ± 0.9^†‡^	1.3 ± 0.9	1.3 ± 0.9^§^	1.2 ± 0.9
Treatment					
Pirfendone	2,937 (28.8)	1,327 (23.8)*^†^	1,610 (34.9)	1,211 (38.3)^§^	399 (27.5)
Median duration, month (IQR)	12.2 (4.2–22.7)	11.7 (4–22)	12.8 (4.4–23.3)	13.5 (4.9–24)	10.4 (3.7–22)
Corticosteroid	6,207 (61.0)	3,510 (63.0)*^†‡^	2,697 (58.5)	1,878 (59.4)^§^	819 (56.5)
Home oxygen supply^‡^	98 (1.0)	46 (0.8)^†^	52 (1.1)	35 (1.1)	17 (1.2)

Data were expressed as mean ± standard deviation or number (%). IPF, idiopathic pulmonary fibrosis; BMI, body mass index; NTM, nontuberculous mycobacterial; COPD, chronic obstructive lung disease; CCI, Charlson comorbidity index; IQR, interquartile range

*The *p*-value was < 0.05 when comparing never and ever smokers. †The p-value was < 0.05 when comparing between never and former smokers. ‡The *p*-value was < 0.05 when comparing between never and current smokers. §The *p*-value was < 0.05 when comparing former and current smokers

Never smokers were older, more frequently female, and less likely to use pirfenidone than ever smokers. Never smokers exhibited higher mortality rates than ever, former, and current smokers (Additional file 1: Table S1). Never smokers also showed higher rates of all-cause or respiratory hospitalisation than ever and former smokers. Current smokers were younger, more frequently male, more likely to require medical aid, and less likely to be prescribed pirfenidone than former smokers (Table 1). Current smokers had lower mortality, longer hospitalisation-free survival time than former smokers (Additional file 1: Table S1).

### Association with all-cause mortality

**Fig. 1:**
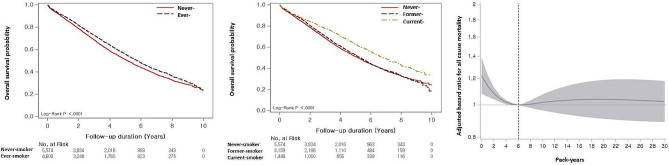
Association between smoking and mortality according to smoking status in patients with IPF. (**a**) Comparison of the survival curves between never and ever smokers in patients with IPF. (**b**) Comparison of the survival curves among never, former, and current smokers in patients with IPF. (**c**) Spline curve analysis of smoking amount for the prognosis The Kaplan–Meier method was used for overall survival estimates, and the log-rank test was used for survival differences by subgroups. The spline curve hazard ratio was computed by adjusting covariates including age, sex, diagnosis year, Charlson comorbidity index, medication (use of steroid and pirfenidone), medical aid, residential address, and low household income. The reference point (6 pack-years) was indicated by a vertical dashed line

During the follow-up (median: 3.1 years; interquartile range: 0.0–10.0 years), 4,576 (44.9%) patients died. Median survival was 5.4 years (95% confidence interval [CI]: 5.3–5.6 years). Never smokers had worse survival (median survival period: 5.1 vs. 5.8 years; *p* < 0.001) than ever smokers (Fig. 1a). Current smokers showed better survival (6.8 years) than never smokers (5.1 years, *p* < 0.001) and former smokers (5.3 years, *p* < 0.001). However, former smokers showed no difference from never (*p* = 0.125) (Fig. 1b).

Cox proportional analysis found that ever smokers were associated with decreased mortality risk in unadjusted and adjusted (HR: 0.853; 95% CI: 0.798–0.912) models compared with never smokers (Table 2). Current smokers were associated with a decreased risk of mortality compared with never smokers in the unadjusted model; however, in the multivariable analysis, both former smokers (HR: 0.926; 95% CI: 0.862–0.996) and current smokers (HR: 0.709; 95% CI: 0.643–0.782) were independently associated with a decreased risk of mortality. The increased number of pack-years showed a marginal association with mortality in unadjusted and adjusted models (HR: 0.998; 95% CI: 0.997–1.000) (Table 2). When smoking amounts were categorised into quartiles, all quartiles showed a decreased risk of mortality compared to never smokers (reference: 0 pack-years) in the unadjusted and multivariable analyses.

**Table 2:** Cox proportional hazards analysis for risk factors of mortality in patients with IPF

	Unadjusted			Multivariable		
	HR	95% CI	*p*-value	HR	95% CI	*p*-value
Smoking status						
Two groups						
Never (*n* = 5,574)	1.000			1.000		
Ever (*n* = 4,608)	0.877	0.827–0.930	< 0.001	0.853	0.798–0.912	< 0.001
Three groups						
Never (*n* = 5,574)	1.000			1.000		
Former (*n* = 3,159)	0.963	0.902–1.027	0.249	0.926	0.862–0.996	0.038
Current (*n* = 1,449)	0.716	0.653–0.785	< 0.001	0.709	0.643–0.782	< 0.001
Smoking amount*	0.998	0.997–1.000	0.033	0.998	0.997–1.000	0.037
Never (*n* = 5,574)	1.000			1.000		
Q1 (*n* = 1,145)	0.870	0.789–0.960	0.005	0.818	0.739–0.906	< 0.001
Q2 (*n* = 1,077)	0.880	0.795–0.973	0.013	0.840	0.755–0.934	0.001
Q3 (*n* = 956)	0.822	0.738–0.915	< 0.001	0.875	0.782–0.979	0.020
Q4 (*n* = 1,430)	0.917	0.842–0.999	0.048	0.878	0.802–0.962	0.005

IPF, idiopathic pulmonary fibrosis; HR, hazard ratio; CI, confidence interval; pyrs, pack-years

*Smoking amount (pack-years) was treated as a continuous variable for the analysis. Smoking amount was divided into Q1 (1–17 pack-years), Q2 (18–29 pack-years), Q3 (30–39 pack-years), and Q4 (40–200 pack-years). The multivariable Cox model was adjusted for age, sex, diagnosis year, Charlson comorbidity index, medication (use of steroid and pirfenidone), medical aid, residential address, and low household income

After adjusting for all covariates (age, sex, diagnosis year, CCI, medication, medical aid, regional types, and low household income), spline HR curve analysis showed a non-linear association between smoking amount and all-cause mortality, with a nadir of mortality risk at 6 pack-years (Fig. 1c). A significant increase in mortality risk was observed below 6 pack-years (highest mortality at 0 pack-years [HR: 1.218; 95% CI: 1.077–1.377]), whereas no increases in mortality risk were found above 6 pack-years when 6 pack-years were used as a reference.

### Association with hospitalisation

**Fig. 2:**
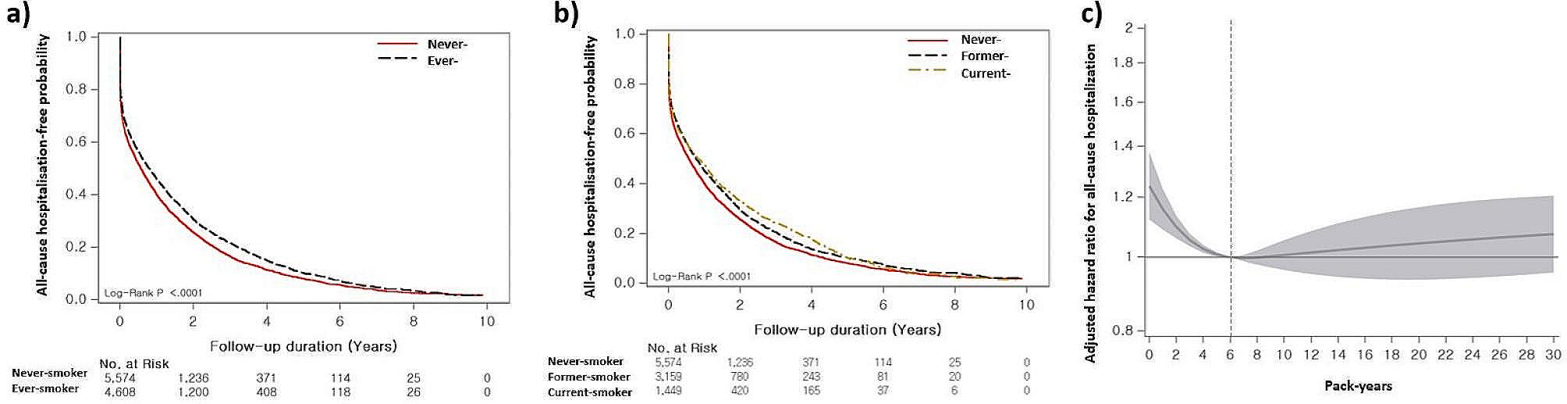
Association between smoking and all-cause hospitalisation according to smoking status in patients with IPF. (**a**) Comparison of the all-cause hospitalisation-free survival curves between never and ever smokers in patients with IPF. (**b**) Comparison of the all-cause hospitalisation-free survival curves among never, former, and current smokers in patients with IPF. (**c**) Spline curve analysis of smoking amount for the all-cause hospitalisation The Kaplan–Meier method was used for all-cause hospitalisation estimates, and the log-rank test was used for all-cause hospitalisation differences by subgroups. The spline curve hazard ratio was computed by adjusting covariates including age, sex, diagnosis year, Charlson comorbidity index, medication (use of steroid and pirfenidone), medical aid, residential address, and low household income. The reference point (6 pack-years) was indicated by a vertical dashed line

**Fig. 3:**
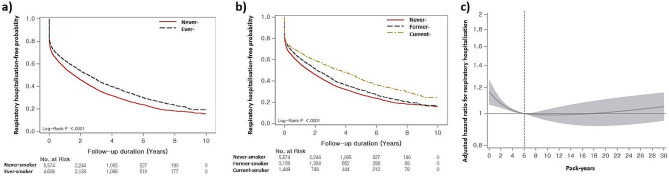
Association between smoking and respiratory hospitalisation according to smoking status in patients with IPF. (**a**) Comparison of the respiratory hospitalisation-free survival curves between never and ever smokers in patients with IPF. (**b**) Comparison of the respiratory hospitalisation-free survival curves among never, former, and current smokers in patients with IPF. (**c**) Spline curve analysis of smoking amount for the respiratory hospitalisation The Kaplan–Meier method was used for respiratory hospitalisation estimates, and the log-rank test was used for respiratory hospitalisation differences by subgroups. Spline curve hazard ratio was computed by adjusting covariates including age, sex, diagnosis year, Charlson comorbidity index, medication (use of steroid, pirfenidone), medical aid, residential address, and low household income. The reference point (6 pack-years) was indicated by a vertical dashed line

The median hospitalisation-free survival time for all-cause and respiratory-cause hospitalisation was 0.7 years (95% CI: 0.6–0.7 years) and 2.0 years (95% CI: 1.9–2.1 years), respectively. In the Kaplan–Meier survival analysis, never smokers had worse all-cause hospitalisation-free survival (median survival period: 0.6 vs. 0.8 years, *p* = 0.001) than ever smokers (Fig. 2a). When classified into three groups, never smokers had worse hospitalisation-free survival (0.6 vs. 0.8 years [former] vs. 0.8 years [current], *p* < 0.001) than the other groups; however, there were no differences between former and current smokers (*p* = 0.747) (Fig. 2b). Regarding respiratory hospitalisation, never smokers showed worse hospitalisation-free survival (median survival period: 1.6 vs. 2.5 years, *p* < 0.001) than ever smokers (Fig. 3a). Never smokers also had worse hospitalisation-free survival (1.6 vs. 2.1 years [former] vs. 3.6 years [current], *p* < 0.001) than former and current smokers (Fig. 3b). Additionally, former smokers had shorter hospitalisation-free survival than current smokers (*p* = 0.001).

In terms of all-cause hospitalisation, ever smokers had a decreased risk of hospitalisation compared with never smokers in the unadjusted and multivariable analysis (HR: 0.901; 95% CI: 0.857–0.974) (Table 3). Former smokers (HR: 0.909; 95% CI: 0.862–0.959) and current smokers (HR: 0.884; 95% CI: 0.827–0.945) were associated with a decreased risk of hospitalisation on the multivariable analysis. In terms of respiratory hospitalisation, ever smokers had a reduced risk of hospitalisation compared with the never smokers in both the unadjusted analysis and adjusted analysis (HR: 0.860; 95% CI: 0.813–0.910) (Table 3). Former smokers (HR: 0.939; 95% CI: 0.883–0.997) and current smokers (HR: 0.746; 95% CI: 0.688–0.808) were independently associated with a reduced risk of respiratory hospitalisation in the multivariable analysis.

**Table 3:** The Cox proportional hazards analysis for the risk factors of hospitalisation in patients with IPF

	All-cause						Respiratory					
	Unadjusted			Multivariable			Unadjusted			Multivariable		
	HR	95% CI	*p*-value	HR	95% CI	*p*-value	HR	95% CI	*p*-value	HR	95% CI	*p*-value
Smoking status												
Two groups												
Never (*n* = 5,574)	1.000			1.000			1.000			1.000		
Ever (*n* = 4,608)	0.854	0.810–0.890	< 0.001	0.901	0.857–0.947	< 0.001	0.809	0.771–0.848	< 0.001	0.860	0.813–0.910	< 0.001
Three groups												
Never (*n* = 5,574)	1.000			1.000			1.000			1.000		
Former (*n* = 3,159)	0.864	0.825–0.905	< 0.001	0.909	0.862–0.959	< 0.001	0.885	0.839–0.992	< 0.001	0.939	0.883–0.997	0.040
Current (*n* = 1,449)	0.833	0.783–0.885	< 0.001	0.884	0.827–0.945	< 0.001	0.695	0.644–0.749	< 0.001	0.746	0.688–0.808	< 0.001
Smoking amount*	0.998	0.997–1.000	0.005	1.000	0.999–1.001	0.989	0.994	0.992–0.995	< 0.001	0.996	0.994–0.998	< 0.001
Never (*n* = 5,574)	1.000			1.000			1.000			1.000		
Q1 (*n* = 1,145)	0.862	0.805–0.924	< 0.001	0.904	0.840–0.972	0.007	0.790	0.728–0.856	< 0.001	1.197	0.957–1.497	0.115
Q2 (*n* = 1,077)	0.818	0.761–0.879	< 0.001	0.874	0.809–0.943	< 0.001	0.796	0.733–0.865	< 0.001	0.830	0.762–0.903	< 0.001
Q3 (*n* = 956)	0.862	0.800–0.929	< 0.001	0.941	0.869–1.108	0.131	0.851	0.781–0.927	< 0.001	0.853	0.782–0.931	< 0.001
Q4 (*n* = 1,430)	0.937	0.881–0.997	0.041	0.988	0.924–1.057	0.730	0.843	0.785–0.906	< 0.001	0.925	0.845–1.103	0.092

IPF, idiopathic pulmonary fibrosis; HR, hazard ratio; CI, confidence interval; pyrs, pack-years

*Smoking amount (pack-years) was treated as a continuous variable for the analysis. Smoking amount was divided into Q1 (1–17 pack-years), Q2 (18–29 pack-years), Q3 (30–39 pack-years), and Q4 (40–200 pack-years). An multivariable model was adjusted for age, sex, diagnosis year, Charlson comorbidity index, medication (use of steroid and pirfenidone), medical aid, residential address, and low household income

In the multivariable analysis, smoking amount was insignificantly associated with all-cause hospitalisation but significantly associated with respiratory hospitalisation (HR: 0.996; 95% CI: 0.994–0.998). When smoking amounts were categorised into quartiles, Q1 (HR: 0.904; 95% CI: 0.840–0.972) and Q2 (HR: 0.874; 95% CI: 0.809–0.943) showed a decreased risk of all-cause hospitalisation compared with never smokers (zero pack-years) (Table 3). A decreased risk of respiratory hospitalisation was also observed in Q2 (HR: 0.835; 95% CI: 0.782–0.892) and Q3 (HR: 0.887; 95% CI: 0.830–0.949) in the multivariable analysis (Table 3).

The spline HR curve analysis, after adjusting all clinical and socioeconomic covariates, showed a non-linear association between smoking amounts and all-cause hospitalisation. Below 6 pack-years, the risk of all-cause hospitalisation significantly increased with the highest risk at zero pack-years (HR: 1.163; 95% CI: 1.065–1.270); however, there was no significant association beyond 6 pack-years (Fig. 2c). Similar trends were exhibited in the spline HR curve analysis for respiratory hospitalisation, with an increased risk below 6 pack-years (Fig. 3c).

### Subgroup analysis stratified by sex and age

We performed stratified analyses by sex. In men, similar to the main analysis, ever smoker or former and current smokers were independently associated with a lower risk of mortality and all-cause or respiratory hospitalisation in the multivariable analysis (Additional file 1: Table S2). In the analysis of smoking amount, high smoking levels were also independently associated with a decreased risk of mortality and respiratory hospitalisation in the multivariable analysis (Additional file 1: Table S3). In women, ever smokers and smoking amount showed a numerical trend towards lower risk of death and hospitalisation, but were only significantly associated with respiratory hospitalisation (Additional file 1: Table S2 and Table S3). Current smokers also showed a reduced risk of all-cause and respiratory hospitalisation in the multivariable analysis (Additional file 1: Table S2).

In the analysis stratified by age group (< 65 years and ≥ 65 years), associations between smoking status and clinical outcomes were consistently observed in the multivariable analysis (Additional file 1: Table S4). In younger age group, ever and current smokers were independently associated with a lower risk of mortality and all-cause or respiratory hospitalisation in the multivariable analysis (Additional file 1: Table S4). In older age group, ever and current smokers were also independently associated with a lower risk of mortality and respiratory hospitalisation in the multivariable analysis (Additional file 1: Table S4). Smoking amount showed a numerical trend towards lower risk of death and hospitalisation, but did not reach statistical significance in either age group (Additional file 1: Table S5).

### Subgroup analysis stratified by years of diagnosis and treatment status

When we performed stratified analyses by quartiles of the year of diagnosis, the results were similar to the main findings (Additional file 1: Table S6). Both ever smokers and current smokers showed a significant association with reduced mortality in the multivariate analysis, with the exception of the third quartile (2013–2014). Respiratory admissions also showed similar results in both groups. However, for former smokers, the results were not significant in most periods except Q4.

In an analysis stratifying patients based on antifibrotis availability (before vs. after October 2015), consistent results were observed, indicating a favourable prognosis for ever smokers compared with never smokers in both time periods (Additional file 1: Table S7). In the analysis stratified by treatment status, the results were also consistent with the main analysis (Additional file 1: Table S8). In the untreated patient group, both ever and current smokers had a significantly lower risk of respiratory hospitalisation. In the group treated with steroids alone, both ever and current smokers also had a reduced risk of mortality, and respiratory hospitalisation. Among patients treated with both steroid and pirfenidone, current smokers had a significant reduction in mortality and respiratory hospitalisation. In the group treated with pirfenidone alone, there was also a trend towards a reduced risk of death in both current and former smokers, although this was not statistically significant.

## Discussion

In this large-scale population-based study using a claim database, we demonstrated an association between smoking status and clinical outcomes in patients with IPF. Current and former smokers had better prognoses, including lower mortality and fewer hospitalisations, than never smokers. We also found a non-linear association between smoking amount and prognosis in IPF.

In our study, smoking was associated with IPF mortality, consistent with previous studies [15, 16, 20]. Kishaba et al. in a retrospective IPF cohort (n = 98) showed that never smokers had worse median survival (18.5 vs. 26.3 months) than ever smokers after adjusting for composite physiologic index (CPI) [15]. King et al. reported that current smokers, being generally younger (54.7 years [current] vs. 62.3 years [never] vs. 62.6 years [former], *p* < 0.05) had better survival (median survival period: 116.4 months) than other groups (former smokers: 25.3 months; never smokers: 27.2 months, *p* < 0.001) in a prospective IPF cohort (n = 238) [16]. Better survival for current smokers may result from earlier diagnoses due to smoking-related symptoms, leading to lead-time bias (healthy smoker’ effect) [29]. This effect can be taken into account in our study because the average age of never-smokers is higher than that of smokers (ever, current and former). However, in our study, both age-adjusted multivariable results and analyses stratified by age showed better prognosis in smokers.

However, Antonious et al. in a retrospective IPF cohort (*n* = 249), reported that current smokers had a lower risk of mortality in an unadjusted analysis than the other groups, but did not show a difference (HR: 0.75; 95% CI: 0.40–1.43) in mortality risk compared with former smokers after adjusting for CPI [17]. In addition, never smokers showed a lower mortality rate in both the unadjusted and CPI-adjusted analyses (HR: 0.48; 95% CI: 0.32–0.71) compared with former smokers [17]. Kärkkäinen et al. in a retrospective IPF cohort (*n* = 128) also demonstrated that current smokers (HR: 0.52; 95% CI: 0.29–0.95) and never smokers (HR: 0.64; 95% CI: 0.42–0.97) had a decreased risk of mortality compared with former smokers in the unadjusted analysis; but this effect disappeared when adjusting for disease severity [18]. Therefore, previous studies have demonstrated that the beneficial effects of smoking on IPF outcomes disappeared when controlling for lung function or disease severity [17, 18]. However, in our study, current smokers had a better prognosis than never or former smokers even after adjusting for individual and socioeconomic variables.

We observed an association between ever smokers and a decreased risk of hospitalisation. Acute exacerbation (AE) is one of the leading causes of hospitalisation in patients with IPF [30]. The effect of smoking on AE occurrence in IPF varies across studies, with some studies showing a positive association [31, 32], whereas others show a negative one [15, 20, 31]. Cao et al., in 107 patients with IPF, demonstrated that ever-smoking was a risk factor for AE occurrence (HR: 1.974; 95% CI: 1.140–3.419) in those with a UIP pattern on high-resolution CT but not in those with a possible UIP pattern [31]. Similarly, the phase 3 trial of nintedanib for patients with IPF (*n* = 1,062) suggested that ever-smoking was associated with increased risk of AE (adjusted HR: 2.13; 95% CI: 0.89–5.13; *p* = 0.09) [32]. However, Song et al., in 461 patients with IPF, reported that ever-smoking was associated with a decreased risk of AE (HR: 0.585; 95% CI: 0.342–1.001, *p* = 0.050) when adjusted by age and lung function [20]. Kishaba et al. also showed that never smokers had a higher AE incidence (50% vs. 18%). than ever smokers [15]. These findings were in line with our results.

This finding of better outcomes in smokers may be partly explained by smoking-induced upregulation of heat shock proteins (HSPs), particularly HSP70 [33]. HSP70, known for its protective effects, including inhibition of transforming growth factor-beta-β (TGF-β)-dependent epithelial-mesenchymal transition and anti-inflammatory properties [34], may delay IPF progression. In addition, smoking has been reported to upregulate autophagy markers, such as microtubule-associated protein 1 A/1B light chain 3B (LC3B) [35]. Increased LC3B activity, known for its antifibrotic effects in alveolar epithelial cells, may also contribute to slower IPF progression [36]. This is supported by an in vitro study showing that inhibition of autophagy by LC3B knockdown in human lung fibroblasts increased α-smooth muscle actin and type I collagen expression, which was further enhanced by TGF-β [37]. In addition, lower levels of LC3B in IPF lung compared to the normal lung [38] highlight its potential role in IPF progression.

Our study showed that a significant association between smoking and favourable prognosis was more frequently observed in men. This may be due to several factors, including physiological differences between the sexes, such as women having anatomically narrower airways and lower lung function [39]. In addition, the potential of smoking to increase oestrogen levels in women may increase the risk of mortality by increasing the likelihood of cancer and thrombosis [40]. Moreover, genetic factors may affect the effects of smoking differently in men and women. A study by Paul showed more smoking-induced genetic changes in female smokers than in males, suggesting a greater susceptibility of women to tobacco carcinogens [41]. However, it is also possible that the relatively smaller sample size of female IPF patients in our study may have resulted in less statistical significance in the observed results.

Our study has some limitations. First, our reliance on medical check-up data may introduce selection bias owing to the inclusion of patients with high health concerns or socioeconomic status. However, the wide coverage of the national health examination in Korea ensures that the characteristics of our study population are representative of the broader IPF population in South Korea. Second, IPF diagnoses may be overestimated owing to the use of diagnostic codes to define IPF cases. Therefore, we utilised both IPF and RID registration codes to define cases. Finally, our analysis was based on a claims database, which limits the inclusion of lung function in the analysis. However, we included clinical variables known to be associated with IPF prognosis, including home oxygen use, medications, and CCI, in our multivariable analysis.

In conclusion, our results suggest that smoking may be associated with clinical outcomes in IPF, with a non-linear association with smoking amount. These findings highlight the complex relationship between smoking and IPF prognosis.

### Electronic supplementary material

Below is the link to the electronic supplementary material.

Additional file 1. Fig. S1 Flow diagram of the study population; Fig. S2 Distribution of smoking pack-years in the IPF cohort; Table S1. Comparison of clinical outcome in patients with IPF according to smoking status; Table S2. The multivariable Cox proportional hazards analysis for the risk factors of prognosis in patients with IPF according to the smoking status stratified by sex; Table S3. The multivariable Cox proportional hazards analysis for the risk factors of prognosis in patients with IPF according to the smoking amount stratified by sex; Table S4. The multivariable Cox proportional hazards analysis for the risk factors of prognosis in patients with IPF stratified by age; Table S5. The multivariable Cox proportional hazards analysis for the risk factors of prognosis in patients with IPF according to the smoking amount stratified by age.; Table S6. The multivariable Cox proportional hazards analysis for the risk factors of prognosis in patients with IPF stratified by year of diagnosis; Table S7. The multivariable Cox proportional hazards analysis for the risk factors of prognosis in patients with IPF stratified by antifibrotics availability; Table S8. The multivariable Cox proportional hazards analysis for the risk factors of prognosis in patients with IPF stratified by the treatment status

## Data Availability

The datasets used and/or analyzed during the current study are available from the corresponding author on reasonable request.

## Abbreviations

IPF: Idiopathic pulmonary fibrosis
CT: Computed tomography
ILD: Intestinal lung disease
NHISS: National Health Insurance Sharing Service
NHI: National Health Insurance
RID: Rare intractable diseases
KCD: Korean Standard Classification of Disease
HR: Hazard ratio
CI: Confidence interval
AE: Acute exacerbation
UIP: Usual interstitial pneumonia
CPI: Composite physiologic index
