# Supplementary material for: Smoking status and clinical outcome in idiopathic pulmonary fibrosis: a nationwide study
Source: Respir Res. 2024 Apr 29;25:191. doi: 10.1186/s12931-024-02819-w (PMC11059669; doi:10.1186/s12931-024-02819-w)
Supplement: Supplementary file 1 — Additional file 1. Fig. S1 Flow diagram of the study population; Fig. S2 Distribution of smoking pack-years in the IPF cohort; Table S1. Comparison of clinical outcome in patients with IPF according to smoking status; Table S2. The multivariable Cox proportional hazards analysis for the risk factors of prognosis in patients with IPF according to the smoking status stratified by sex; Table S3. The multivariable Cox proportional hazards analysis for the risk factors of prognosis in patients with IPF according to the smoking amount stratified by sex; Table S4. The multivariable Cox proportional hazards analysis for the risk factors of prognosis in patients with IPF stratified by age; Table S5. The multivariable Cox proportional hazards analysis for the risk factors of prognosis in patients with IPF according to the smoking amount stratified by age.; Table S6. The multivariable Cox proportional hazards analysis for the risk factors of prognosis in patients with IPF stratified by year of diagnosis; Table S7. The multivariable Cox proportional hazards analysis for the risk factors of prognosis in patients with IPF stratified by antifibrotics availability; Table S8. The multivariable Cox proportional hazards analysis for the risk factors of prognosis in patients with IPF stratified by the treatment status [file 12931_2024_2819_MOESM1_ESM.docx]

**Smoking status and clinical outcome in idiopathic pulmonary fibrosis: a nationwide study**

Hee-Young Yoon^1^, Hoseob Kim^2^, Yoonjong Bae^2^, Jin Woo Song^3^

^1^Division of Allergy and Respiratory Diseases, Soonchunhyang University Seoul Hospital, Seoul, Republic of Korea

^2^Department of Data Science, Hanmi Pharm. Co., Ltd., Seoul, Republic of Korea

^3^Department of Pulmonary and Critical Care Medicine, Asan Medical Center, University of Ulsan College of Medicine, Seoul, Republic of Korea

**Table S1**. Comparison of clinical outcome in patients with IPF according to smoking status

|  | **Total** | **Never** | **Ever smokers** | | |
| --- | --- | --- | --- | --- | --- |
|  |  |  | **Total** | **Former** | **Current** |
| Number of patients | 10,182 | 5,574 | 4,608 | 3,159 | 1,449 |
| Death |  |  |  |  |  |
| Number of events | 4,576 (44.9) | 2,629 (47.2)*^†‡^ | 1947 (42.3) | 1,398 (44.3)^§^ | 549 (37.9) |
| Time death, median years (IQR) | 3.1 (0.0–10.0) | 3.0 (0.0–10.0)*^‡^ | 3.1 (0.0–10.0) | 2.9 (0.0–10.0)^§^ | 3.5 (0.0–10.0) |
| All-cause hospitalisation |  |  |  |  |  |
| Number of events | 7.5 ± 10.8 | 8.3 ± 13.8*^†^ | 7.4 ± 11.3 | 7.0 ± 9.8^§^ | 8.1 ± 11.3 |
| Time to admission, median (IQR) | 0.6 (0.0–9.9) | 0.6 (0.0–9.9)*^†‡^ | 0.8 (0.0–9.8) | 0.7 (0.0–9.8)^§^ | 0.8 (0.0–9.8) |
| Respiratory hospitalisation |  |  |  |  |  |
| Number of events | 4.1 ± 22.4 | 4.4 ± 14.9*^†^ | 3.7 ± 6.2 | 3.4 ± 5.8 | 4.2 ± 6.7 |
| Time to admission, median (IQR) | 1.8 (0.0–10.0) | 1.4 (0.0–10.0)*^†‡^ | 1.8 (0.0–10.0) | 1.6 (0.0–10.0)^§^ | 2.2 (0.0–10.0) |

Data were expressed as mean ± standard deviation, number (%), or median (interquartile range). IPF, idiopathic pulmonary fibrosis; IQR, interquartile range

*The p-value was <0.05 when comparing never and ever smokers. †The p-value was <0.05 when comparing between never and former smokers. ‡The p-value was <0.05 when comparing between never and current smokers. §The p-value was <0.05 when comparing former and current smokers.**Table S2.** The multivariable Cox proportional hazards analysis for the risk factors of prognosis in patients with IPF according to the smoking status stratified by sex.

|  | Mortality | | | All-cause hospitalisation | | | Respiratory hospitalisation | | |
| --- | --- | --- | --- | --- | --- | --- | --- | --- | --- |
|  | aHR | 95% CI | p-value | aHR | 95% CI | p-value | aHR | 95% CI | p-value |
| Male (n = 7,528) |  |  |  |  |  |  |  |  |  |
| Two groups |  |  |  |  |  |  |  |  |  |
| Never (n = 3,011) | 1.000 |  |  | 1.000 |  |  | 1.000 |  |  |
| Ever (n = 4,517) | 0.834 | 0.779-0.893 | <0.001 | 0.921 | 0.876-0.969 | 0.002 | 0.869 | 0.820-0.920 | <0.001 |
| Three groups |  |  |  |  |  |  |  |  |  |
| Never (n = 3,011) | 1.000 |  |  | 1.000 |  |  | 1.000 |  |  |
| Former (n = 3,129) | 0.906 | 0.842-0.975 | 0.008 | 0.930 | 0.880-0.982 | 0.009 | 0.931 | 0.87-0.991 | 0.024 |
| Current (n = 1,388) | 0.681 | 0.616-0.753 | <0.001 | 0.901 | 0.840-0.967 | 0.004 | 0.733 | 0.675-0.798 | <0.001 |
| Female (n = 2,654) |  |  |  |  |  |  |  |  |  |
| Two groups |  |  |  |  |  |  |  |  |  |
| Never (n = 2,563) | 1.000 |  |  | 1.000 |  |  | 1.000 |  |  |
| Ever (n = 91) | 0.924 | 0.656-1.301 | 0.651 | 0.876 | 0.699-1.098 | 0.251 | 0.717 | 0.545-0.942 | 0.017 |
| Three groups |  |  |  |  |  |  |  |  |  |
| Never (n = 2,563) | 1.000 |  |  | 1.000 |  |  | 1.000 |  |  |
| Former (n = 30) | 1.732 | 1.019-2.943 | 0.042 | 1.383 | 0.958-1.996 | 0.083 | 0.933 | 0.593-1.469 | 0.765 |
| Current (n = 61) | 0.697 | 0.447-1.085 | 0.110 | 0.720 | 0.542-0.956 | 0.023 | 0.635 | 0.452-0.891 | 0.009 |

IPF, idiopathic pulmonary fibrosis; aHR, adjusted hazard ratio; CI, confidence interval; pyrs, pack-years

An multivariable model was adjusted for age, diagnosis year, Charlson comorbidity index, medication (use of steroid and pirfenidone), medical aid, residential address, and low household income.

**Table S3.** The multivariable Cox proportional hazards analysis for the risk factors of prognosis in patients with IPF according to the smoking amount stratified by sex.

|  | Mortality | | | All-cause hospitalisation | | | Respiratory hospitalisation | | |
| --- | --- | --- | --- | --- | --- | --- | --- | --- | --- |
|  | aHR | 95% CI | p-value | aHR | 95% CI | p-value | aHR | 95% CI | p-value |
| Male (n = 7,528) |  |  |  |  |  |  |  |  |  |
| Smoking amount* | 0.998 | 0.996-1.000 | 0.017 | 1.000 | 0.999-1.001 | 0.958 | 0.999 | 0.997-1.000 | 0.032 |
| Never (n = 3,011) | 1.000 |  |  | 1.000 |  |  | 1.000 |  |  |
| Q1 (n = 1,096) | 0.800 | 0.7200.889 | <0.001 | 0.897 | 0.831-0.967 | 0.005 | 0.835 | 0.765-0.911 | <0.001 |
| Q2 (n = 1,059) | 0.823 | 0.739-0.917 | 0.000 | 0.862 | 0.798-0.932 | 0.000 | 0.841 | 0.769-0.919 | <0.001 |
| Q3 (n = 942) | 0.842 | 0.751-0.944 | 0.003 | 0.933 | 0.8601.011 | 0.092 | 0.915 | 0.835-1.004 | 0.061 |
| Q4 (n = 1,042) | 0.862 | 0.786-0.945 | 0.002 | 0.980 | 0.915-1.049 | 0.563 | 0.888 | 0.821-0.961 | 0.003 |
| Female (n = 2,654) |  |  |  |  |  |  |  |  |  |
| Smoking amount* | 0.992 | 0.978-1.006 | 0.269 | 0.993 | 0.983-1.003 | 0.186 | 0.985 | 0.972-0.997 | 0.016 |
| Never (n = 2,563) | 1.000 |  |  | 1.000 |  |  | 1.000 |  |  |
| Q1 (n = 49) | 1.045 | 0.671-1.628 | 0.845 | 0.931 | 0.6901.256 | 0.640 | 0.653 | 0.446-0.956 | 0.029 |
| Q2 (n = 18) | 0.765 | 0.318-1.843 | 0.551 | 0.868 | 0.522-1.443 | 0.586 | 1.120 | 0.648-1.933 | 0.685 |
| Q3 (n = 14) | 1.524 | 0.682-3.408 | 0.304 | 0.627 | 0.337-1.168 | 0.142 | 0.679 | 0.339-1.360 | 0.275 |
| Q4 (n = 10) | 0.417 | 0.134-1.298 | 0.131 | 1.054 | 0.547-2.031 | 0.875 | 0.542 | 0.225-1.305 | 0.172 |

IPF, idiopathic pulmonary fibrosis; aHR, adjusted hazard ratio; CI, confidence interval; pyrs, pack-years

*Smoking amount (pack-years) was treated as a continuous variable for the analysis.

Smoking amount was divided into Q1 (1–17 pack-years), Q2 (18–29 pack-years), Q3 (30–39 pack-years), and Q4 (40–200 pack-years). An multivariable model was adjusted for age, diagnosis year, Charlson comorbidity index, medication (use of steroid and pirfenidone), medical aid, residential address, and low household income.

**Table S4.** The multivariable Cox proportional hazards analysis for the risk factors of prognosis in patients with IPF according to the smoking status stratified by age

|  | Mortality | | | All-cause hospitalisation | | | Respiratory hospitalisation | | |
| --- | --- | --- | --- | --- | --- | --- | --- | --- | --- |
|  | aHR | 95% CI | p-value | aHR | 95% CI | p-value | aHR | 95% CI | p-value |
| < 65 years (n = 2793) |  |  |  |  |  |  |  |  |  |
| Two groups |  |  |  |  |  |  |  |  |  |
| Never (n = 1,316) | 1.000 |  |  | 1.000 |  |  | 1.000 |  |  |
| Ever (n = 1,477) | 0.821 | 0.705-0.955 | 0.010 | 0.852 | 0.771-0.941 | 0.002 | 0.831 | 0.743-0.930 | 0.001 |
| Three groups |  |  |  |  |  |  |  |  |  |
| Never (n = 1,316) | 1.000 |  |  | 1.000 |  |  | 1.000 |  |  |
| Former (n = 900) | 0.922 | 0.782-1.086 | 0.331 | 0.846 | 0.758-0.944 | 0.003 | 0.913 | 0.807-1.032 | 0.144 |
| Current (n = 577) | 0.675 | 0.555-0.822 | <0.001 | 0.860 | 0.762-0.971 | 0.015 | 0.718 | 0.623-0.827 | <0.001 |
| ≥ 65 years (n = 7,389) |  |  |  |  |  |  |  |  |  |
| Two groups |  |  |  |  |  |  |  |  |  |
| Never (n = 4,258) | 1.000 |  |  | 1.000 |  |  | 1.000 |  |  |
| Ever (n = 3,131) | 0.865 | 0.803-0.931 | <0.001 | 0.954 | 0.902-1.010 | 0.105 | 0.881 | 0.826-0.940 | <0.001 |
| Three groups |  |  |  |  |  |  |  |  |  |
| Never (n = 4,258) | 1.000 |  |  | 1.000 |  |  | 1.000 |  |  |
| Former (n = 2,259) | 0.906 | 0.836-0.982 | 0.017 | 0.966 | 0.908-1.028 | 0.274 | 0.942 | 0.879-1.010 | 0.095 |
| Current (n = 832) | 0.767 | 0.685-0.860 | <0.001 | 0.926 | 0.852-1.006 | 0.067 | 0.745 | 0.674-0.822 | <0.001 |

IPF, idiopathic pulmonary fibrosis; aHR, adjusted hazard ratio; CI, confidence interval; pyrs, pack-years

An multivariable model was adjusted for sex, sex, diagnosis year, Charlson comorbidity index, medication (use of steroid and pirfenidone), medical aid, residential address, and low household income.

**Table S5.** The multivariable Cox proportional hazards analysis for the risk factors of prognosis in patients with IPF according to the smoking amount stratified by age

|  | Mortality | | | All-cause hospitalisation | | | Respiratory hospitalisation | | | |
| --- | --- | --- | --- | --- | --- | --- | --- | --- | --- | --- |
|  | aHR | 95% CI | p-value | aHR | 95% CI | p-value | aHR | 95% CI | p-value | |
| < 65 years (n = 2793) |  |  |  |  |  |  |  |  |  | |
| Smoking amount* | 0.998 | 0.995-1.002 | 0.396 | 0.999 | 0.997-1.001 | 0.395 | 0.997 | 0.995-1.000 | 0.054 | |
| Never (n = 1,316) | 1.000 |  |  | 1.000 |  |  | 1.000 |  |  | |
| Q1 (n = 343) | 0.815 | 0.651-1.019 | 0.073 | 0.865 | 0.751-0.996 | 0.044 | 0.836 | 0.712-0.983 | 0.030 | |
| Q2 (n = 347) | 0.824 | 0.658-1.032 | 0.091 | 0.765 | 0.662-0.883 | <0.001 | 0.805 | 0.684-0.947 | 0.009 | |
| Q3 (n = 380) | 0.845 | 0.682-1.045 | 0.121 | 0.885 | 0.771-1.017 | 0.085 | 0.886 | 0.758-1.037 | 0.133 | |
| Q4 (n = 407) | 0.803 | 0.655-0.984 | 0.035 | 0.892 | 0.780-1.021 | 0.098 | 0.801 | 0.686-0.936 | 0.005 | |
| ≥ 65 years (n = 7,389) |  |  |  |  |  |  |  |  |  | |
| Smoking amount* | 0.998 | 0.997-1.000 | 0.066 | 1.000 | 0.999-1.002 | 0.549 | 0.999 | 0.997-1.001 | 0.216 | |
| Never (n = 4,258) | 1.000 |  |  | 1.000 |  |  | 1.000 |  |  | |
| Q1 (n = 802) | 0.823 | 0.734-0.924 | 0.001 | 0.931 | 0.838-0.995 | 0.038 | 0.822 | 0.743-0.909 | <0.001 | |
| Q2 (n = 730) | 0.867 | 0.768-0.979 | 0.021 | 0.916 | 0.837-1.003 | 0.057 | 0.856 | 0.772-0.950 | 0.004 |  |
| Q3 (n = 576) | 0.858 | 0.750-0.981 | 0.025 | 0.936 | 0.849-1.033 | 0.191 | 0.902 | 0.805-1.010 | 0.074 | |
| Q4 (n = 1,023) | 0.901 | 0.813-0.998 | 0.046 | 1.032 | 0.954-1.116 | 0.431 | 0.940 | 0.860-1.029 | 0.180 | |

IPF, idiopathic pulmonary fibrosis; aHR, adjusted hazard ratio; CI, confidence interval; pyrs, pack-years

*Smoking amount (pack-years) was treated as a continuous variable for the analysis. Smoking amount was divided into Q1 (1–17 pack-years), Q2 (18–29 pack-years), Q3 (30–39 pack-years), and Q4 (40–200 pack-years). An multivariable model was adjusted for sex, diagnosis year, Charlson comorbidity index, medication (use of steroid and pirfenidone), medical aid, residential address, and low household income.

**Table S6.** The multivariable Cox proportional hazards analysis for the risk factors of prognosis in patients with IPF according to the smoking status stratified by year of diagnosis

|  | Mortality | | | All-cause hospitalisation | | | Respiratory hospitalisation | | |
| --- | --- | --- | --- | --- | --- | --- | --- | --- | --- |
|  | aHR | 95% CI | p-value | aHR | 95% CI | p-value | aHR | 95% CI | p-value |
| Q1 (2009-2010) (n = 1,620) | | | | | | | | | |
| Two groups |  |  |  |  |  |  |  |  |  |
| Never (n = 924) | 1.000 |  |  | 1.000 |  |  | 1.000 |  |  |
| Ever (n = 696) | 0.845 | 0.738-0.968 | 0.015 | 0.926 | 0.825-1.040 | 0.195 | 0.883 | 0.777-1.004 | 0.058 |
| Three groups |  |  |  |  |  |  |  |  |  |
| Never (n = 924) | 1.000 |  |  | 1.000 |  |  | 1.000 |  |  |
| Former (n = 457) | 0.928 | 0.801-1.077 | 0.326 | 0.933 | 0.820-1.061 | 0.290 | 0.975 | 0.847-1.122 | 0.726 |
| Current (n = 239) | 0.698 | 0.574-0.848 | <0.001 | 0.914 | 0.783-1.068 | 0.258 | 0.735 | 0.614-0.878 | 0.001 |
| Q2 (2011-2012) (n = 2,089) | | | | | | | | | |
| Two groups |  |  |  |  |  |  |  |  |  |
| Never (n = 1,160) | 1.000 |  |  | 1.000 |  |  | 1.000 |  |  |
| Ever (n = 929) | 0.858 | 0.754-0.976 | 0.020 | Ever |  | 0.936 | 0.893 | 0.795-1.004 | 0.058 |
| Three groups |  |  |  |  |  |  |  |  |  |
| Never (n = 1,160) | 1.000 |  |  | 1.000 |  |  | 1.000 |  |  |
| Former (n = 603) | 0.941 | 0.817-1.083 | 0.398 | 0.935 | 0.832-1.051 | 0.261 | 0.954 | 0.840-1.084 | 0.472 |
| Current (n = 326) | 0.708 | 0.588-0.851 | <0.001 | 0.936 | 0.815-1.075 | 0.348 | 0.788 | 0.671-0.924 | 0.003 |
| Q3 (2013-2014) (n = 2,362) | | | | | | | | | |
| Two groups |  |  |  |  |  |  |  |  |  |
| Never (n = 1,281) | 1.000 |  |  | 1.000 |  |  | 1.000 |  |  |
| Ever (n = 1,081) | 0.884 | 0.775-1.009 | 0.068 | 0.951 | 0.861-1.050 | 0.318 | 0.851 | 0.761-0.951 | 0.005 |
| Three groups |  |  |  |  |  |  |  |  |  |
| Never (n = 1,281) | 1.000 |  |  | 1.000 |  |  | 1.000 |  |  |
| Former (n = 731) | 0.903 | 0.782-1.043 | 0.165 | 0.981 | 0.881-1.093 | 0.731 | 0.931 | 0.825-1.052 | 0.252 |
| Current (n = 350) | 0.844 | 0.698-1.020 | 0.079 | 0.893 | 0.779-1.022 | 0.101 | 0.702 | 0.597-0.825 | <0.001 |
| Q4 (2015-2017) (n = 4,111) | | | | | | | | | |
| Two groups |  |  |  |  |  |  |  |  |  |
| Never (n = 2,209) | 1.000 |  |  | 1.000 |  |  | 1.000 |  |  |
| Ever (n = 1,902) | 0.855 | 0.746-0.980 | 0.025 | 0.907 | 0.836-0.986 | 0.021 | 0.859 | 0.779-0.947 | 0.002 |
| Three groups |  |  |  |  |  |  |  |  |  |
| Never (n = 2,209) | 1.000 |  |  | 1.000 |  |  | 1.000 |  |  |
| Former (n = 1,368) | 0.882 | 0.762-1.020 | 0.090 | 0.904 | 0.827-0.988 | 0.026 | 0.903 | 0.813-1.002 | 0.055 |
| Current (n = 534) | 0.777 | 0.626-0.964 | 0.022 | 0.917 | 0.814-1.033 | 0.156 | 0.747 | 0.644-0.866 | <0.001 |

IPF, idiopathic pulmonary fibrosis; aHR, adjusted hazard ratio; CI, confidence interval

An multivariable model was adjusted for age, sex, Charlson comorbidity index, medication (use of steroid and pirfenidone), medical aid, residential address, and low household income.

**Table S7.** The multivariable Cox proportional hazards analysis for the risk factors of prognosis in patients with IPF according to the smoking status stratified by antifibrotics availability

|  | Mortality | | | All-cause hospitalisation | | | Respiratory hospitalisation | | |
| --- | --- | --- | --- | --- | --- | --- | --- | --- | --- |
|  | aHR | 95% CI | p-value | aHR | 95% CI | p-value | aHR | 95% CI | p-value |
| Before Oct 2015 (n = 6,936) | | | | | | | | | |
| Two groups |  |  |  |  |  |  |  |  |  |
| Never (n = 3,860) | 1.000 |  |  | 1.000 |  |  | 1.000 |  |  |
| Ever (n = 3,076) | 0.880 | 0.818-0.946 | 0.001 | 0.951 | 0.898-1.007 | 0.084 | 0.892 | 0.837-0.952 | <0.001 |
| Three groups |  |  |  |  |  |  |  |  |  |
| Never (n = 3,860) | 1.000 |  |  | 1.000 |  |  | 1.000 |  |  |
| Former (n = 2,071) | 0.944 | 0.872-1.022 | 0.158 | 0.964 | 0.905-1.027 | 0.258 | 0.968 | 0.902-1.038 | 0.357 |
| Current (n = 1,005) | 0.752 | 0.676-0.836 | <0.001 | 0.924 | 0.855-0.999 | 0.048 | 0.755 | 0.689-0.828 | <0.001 |
| After Oct 2015 (n = 3,246) | | | | | | | | | |
| Two groups |  |  |  |  |  |  |  |  |  |
| Never (n = 1,714) | 1.000 |  |  | 1.000 |  |  | 1.000 |  |  |
| Ever (n = 1,532) | 0.782 | 0.665-0.919 | 0.003 | 0.869 | 0.791-0.956 | 0.004 | 0.810 | 0.724-0.907 | <0.001 |
| Three groups |  |  |  |  |  |  |  |  |  |
| Never (n = 1,714) | 1.000 |  |  | 1.000 |  |  | 1.000 |  |  |
| Former (n = 1,088) | 0.789 | 0.664-0.938 | 0.007 | 0.861 | 0.778-0.954 | 0.004 | 0.854 | 0.757-0.963 | 0.010 |
| Current (n = 444) | 0.762 | 0.594-0.978 | 0.033 | 0.890 | 0.778-1.018 | 0.090 | 0.703 | 0.594-0.833 | <0.001 |

IPF, idiopathic pulmonary fibrosis; aHR, adjusted hazard ratio; CI, confidence interval; pyrs, pack-years

An multivariable model was adjusted for age, sex, diagnosis year, Charlson comorbidity index, medication (use of steroid and pirfenidone), medical aid, residential address, and low household income.

**Table S8.** The multivariable Cox proportional hazards analysis for the risk factors of prognosis in patients with IPF according to the smoking status stratified by the treatment status

|  | Mortality | | | All-cause hospitalisation | | | Respiratory hospitalisation | | |
| --- | --- | --- | --- | --- | --- | --- | --- | --- | --- |
|  | aHR | 95% CI | p-value | aHR | 95% CI | p-value | aHR | 95% CI | p-value |
| None (n = 3,182)  Two groups |  |  |  |  |  |  |  |  |  |
| Never (n =1,670) | 1.000 |  |  | 1.000 |  |  | 1.000 |  |  |
| Ever (n =1,512) | 0.976 | 0.859-1.108 | 0.705 | 0.967 | 0.882-1.060 | 0.474 | 0.881 | 0.782-0.993 | 0.038 |
| Three groups |  |  |  |  |  |  |  |  |  |
| Never (n = 1,670) | 1.000 |  |  | 1.000 |  |  | 1.000 |  |  |
| Former (n = 932) | 1.024 | 0.889-1.178 | 0.744 | 0.982 | 0.887-1.088 | 0.729 | 0.934 | 0.818-1.067 | 0.313 |
| Current (n = 580) | 0.896 | 0.756-1.062 | 0.207 | 0.944 | 0.840-1.061 | 0.334 | 0.798 | 0.680-0.936 | 0.006 |
| Only steroid (n = 3,714)  Two groups |  |  |  |  |  |  |  |  |  |
| Never (n = 2,237) | 1.000 |  |  | 1.000 |  |  | 1.000 |  |  |
| Ever (n = 1,477) | 0.898 | 0.821-0.983 | 0.020 | 0.941 | 0.870-1.017 | 0.122 | 0.923 | 0.850-1.001 | 0.054 |
| Three groups |  |  |  |  |  |  |  |  |  |
| Never (n = 2,237) | 1.000 |  |  | 1.000 |  |  | 1.000 |  |  |
| Former (n = 1,018) | 0.964 | 0.873-1.063 | 0.460 | 0.946 | 0.868-1.030 | 0.203 | 0.969 | 0.886-1.060 | 0.489 |
| Current (n = 459) | 0.763 | 0.666-0.873 | <0.001 | 0.929 | 0.833-1.037 | 0.189 | 0.830 | 0.737-0.934 | 0.002 |
| Only pirfenidone (n = 1,598)  Two groups |  |  |  |  |  |  |  |  |  |
| Never (n = 775) | 1.000 |  |  | 1.000 |  |  | 1.000 |  |  |
| Ever (n = 823) | 0.719 | 0.517-1.001 | 0.051 | 0.972 | 0.850-1.112 | 0.683 | 0.881 | 0.743-1.046 | 0.148 |
| Three groups |  |  |  |  |  |  |  |  |  |
| Never (n = 775) | 1.000 |  |  | 1.000 |  |  | 1.000 |  |  |
| Former (n = 598) | 0.714 | 0.501-1.018 | 0.063 | 0.921 | 0.797-1.064 | 0.264 | 0.897 | 0.747-1.077 | 0.243 |
| Current (n = 225) | 0.736 | 0.442-1.225 | 0.238 | 1.121 | 0.931-1.351 | 0.227 | 0.841 | 0.657-1.077 | 0.171 |
| Both (n = 1,688)  Two groups |  |  |  |  |  |  |  |  |  |
| Never (n = 892) | 1.000 |  |  | 1.000 |  |  | 1.000 |  |  |
| Ever (n = 796) | 0.869 | 0.728-1.038 | 0.122 | 0.936 | 0.833-1.052 | 0.269 | 0.960 | 0.849-1.086 | 0.520 |
| Three groups |  |  |  |  |  |  |  |  |  |
| Never (n = 892) | 1.000 |  |  | 1.000 |  |  | 1.000 |  |  |
| Former (n = 611) | 0.945 | 0.787-1.136 | 0.549 | 0.955 | 0.844-1.080 | 0.461 | 1.030 | 0.904-1.172 | 0.656 |
| Current (n = 185) | 0.610 | 0.444-0.838 | 0.002 | 0.877 | 0.733-1.049 | 0.150 | 0.771 | 0.636-0.935 | 0.008 |

IPF, idiopathic pulmonary fibrosis; aHR, adjusted hazard ratio; CI, confidence interval; pyrs, pack-years

An multivariable model was adjusted for age, sex, diagnosis year, Charlson comorbidity index, medical aid, residential address, and low household income.

**Fig. S1** Flow diagram of the study population


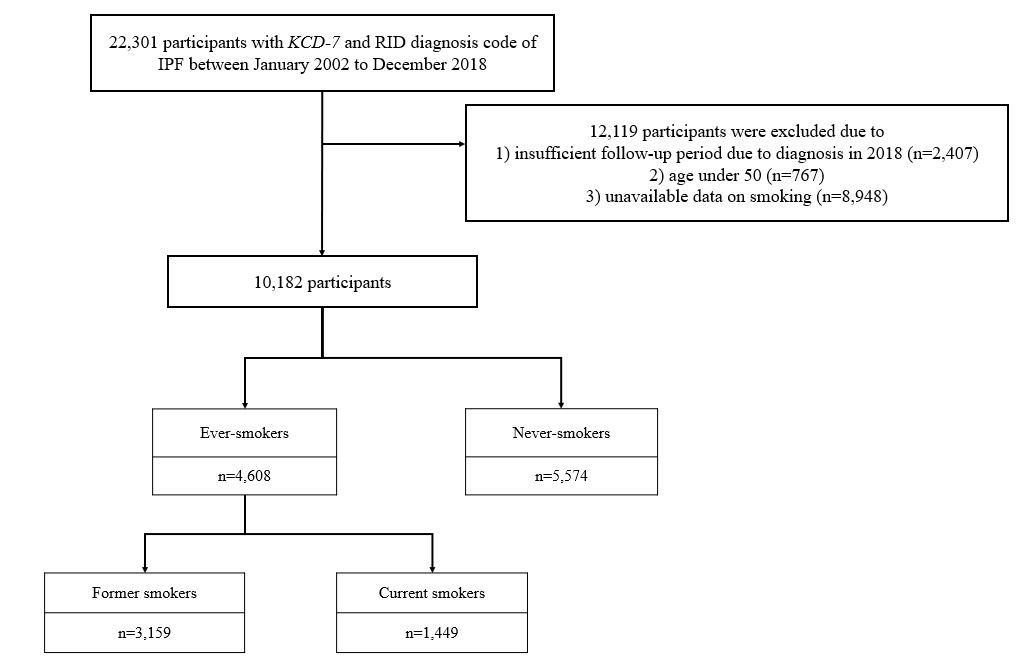


IPF, idiopathic pulmonary fibrosis; KCD-7, Korean Standard Classification of Diseases, 7th edition; RID, rare intractable diseases

**Fig. S2** Distribution of smoking pack-years in the IPF cohort


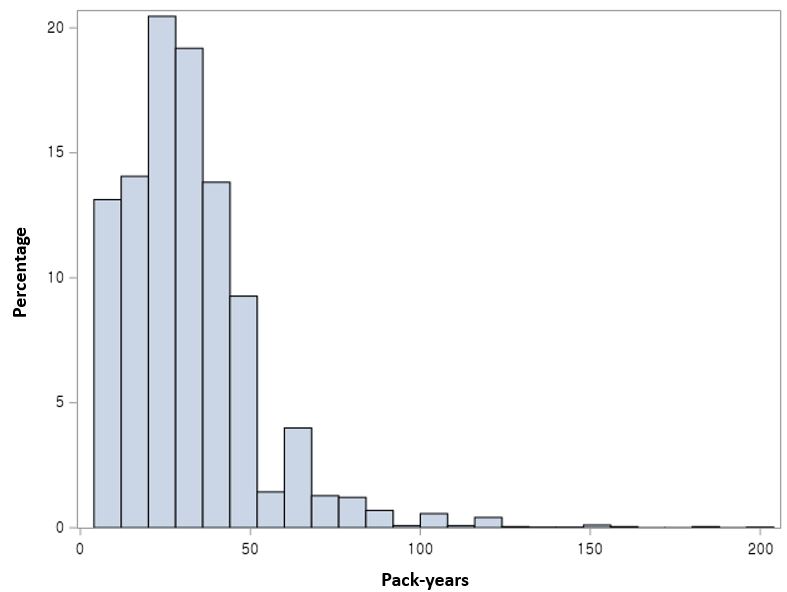
IPF, idiopathic pulmonary fibrosis

The x-axis represents intervals of 10 pack-years, while the y-axis represents the percentage of patients within each interval among the entire study population.
